# When a rare simultaneous infection simulates a local extension of a rectal cancer: a case report

**DOI:** 10.1186/s13256-021-02989-x

**Published:** 2021-08-12

**Authors:** Hicham Baba, Jawad Fassi Fihri, Mohammed Essaid Ramraoui, Ahmed Elguazzar, Ahmed Zeroual, Mohammed Lahkim, Ahmed El Khader, Abdessamad Achour, Rachid El Barni

**Affiliations:** 1 Department of General Surgery, Avicenna Military Hospital, Marrakesh, Morocco; 2 Faculty of Medecine and Pharmacy, Cadi Ayyad University, Marrakesh, Morocco

**Keywords:** Actinomycosis, Adenocarcinoma, Mesorectum

## Abstract

**Background:**

Actinomycosis is a rare chronic infection caused by *Actinomyces*. The abdominopelvic localization of this pathology makes preoperative diagnosis extremely exceptional. The following report is the case of a patient treated for adenocarcinoma of the middle rectum associated with mesorectal actinomycosis. The diagnosis of actinomycosis was made postoperatively.

**Case presentation:**

A 69-year-old Caucasian male patient was admitted for rectal bleeding. Clinical and paraclinical assessment revealed a middle rectum adenocarcinoma with local extension in the mesorectum. The patient underwent an anterior resection of the rectum by laparotomy after neoadjuvant chemoradiotherapy. Postoperative follow-up was simple. Pathological study of the specimen noted complete sterilization of the rectal adenocarcinoma and the presence of large foci of suppurative necrosis containing actinomycotic grains in the mesorectum.

**Conclusion:**

Abdominopelvic actinomycosis is a rare pathology and has therefore rarely been dealt with. This issue can lead to unnecessary and mutilating surgery. We report an exceptional coincidence of rectal adenocarcinoma and mesorectal actinomycosis mistaken for mesorectal extension of the cancer.

## Background

Actinomycosis is a rare chronic granulomatous infection caused by *Actinomyces*, which are saprophytic bacteria in the oral cavity and the digestive tract. The abdominopelvic localization of actinomycosis, often overlooked because of its rarity, poses problems of differential diagnosis especially with neoplastic pathology. The rarity of this pathology is why its diagnosis has often been established on surgical resection pieces. These resections are often mutilating and unnecessary since the treatment is simply based on antibiotics [1].

The following report is the case of a patient with “locally advanced” adenocarcinoma of the middle rectum through which the final histologic examination of the surgical specimen revealed a complete response of the adenocarcinoma to the neoadjuvant chemoradiotherapy associated with mesorectal actinomycosis. The local extension of the cancer in the mesorectum, suspected preoperatively, was nothing other than this synchronous mesorectal actinomycosis.

## Case presentation

A 69-year-old Caucasian male patient with well-balanced type 2 diabetes consulted for rectal bleeding and rectal syndrome. Rectal examination revealed a budding and circumferential tumor at 7 cm of the anal margin; the rest of the clinical examination was normal.

**Fig. 1:**
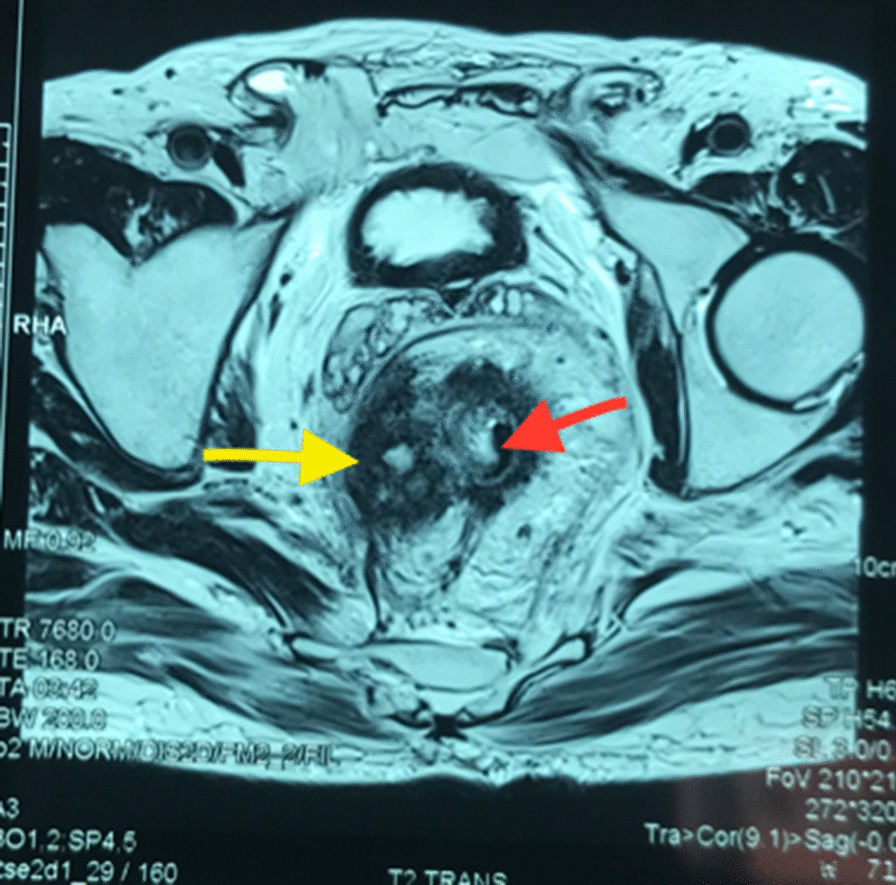
MRI showing the rectal tumor (red arrow) with its “extension” in the mesorectum (yellow arrow)

**Fig. 2:**
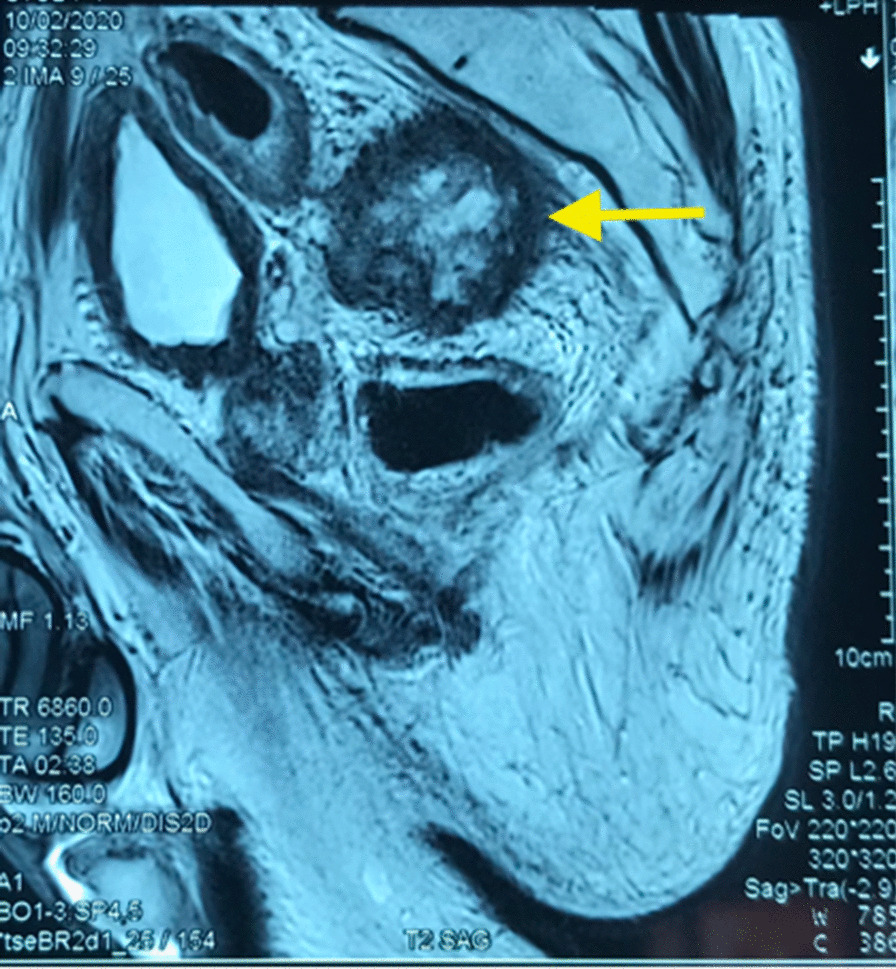
The actinomycotic pseudotumor (yellow arrow)

Colonoscopy revealed ulcerative budding tumor 7 cm from the anal verge; exploration of the rest of the colon showed no other anomalies. Pathological examination of the biopsy revealed a well-differentiated and infiltrating lieberkuhnien adenocarcinoma. Magnetic resonance imaging (MRI) showed a circumferential and irregular parietal thickness of the middle rectum, associated with a heterogeneous mass with exophytic development, infiltrating the mesorectum and arriving at the contact of the mesorectal fascia on the right (Figs. 1,2). Thoracoabdominopelvic CT scan did not show secondary locations. Blood tests noted anemia at 10.8 g/dl without any other abnormality.

**Fig. 3:**
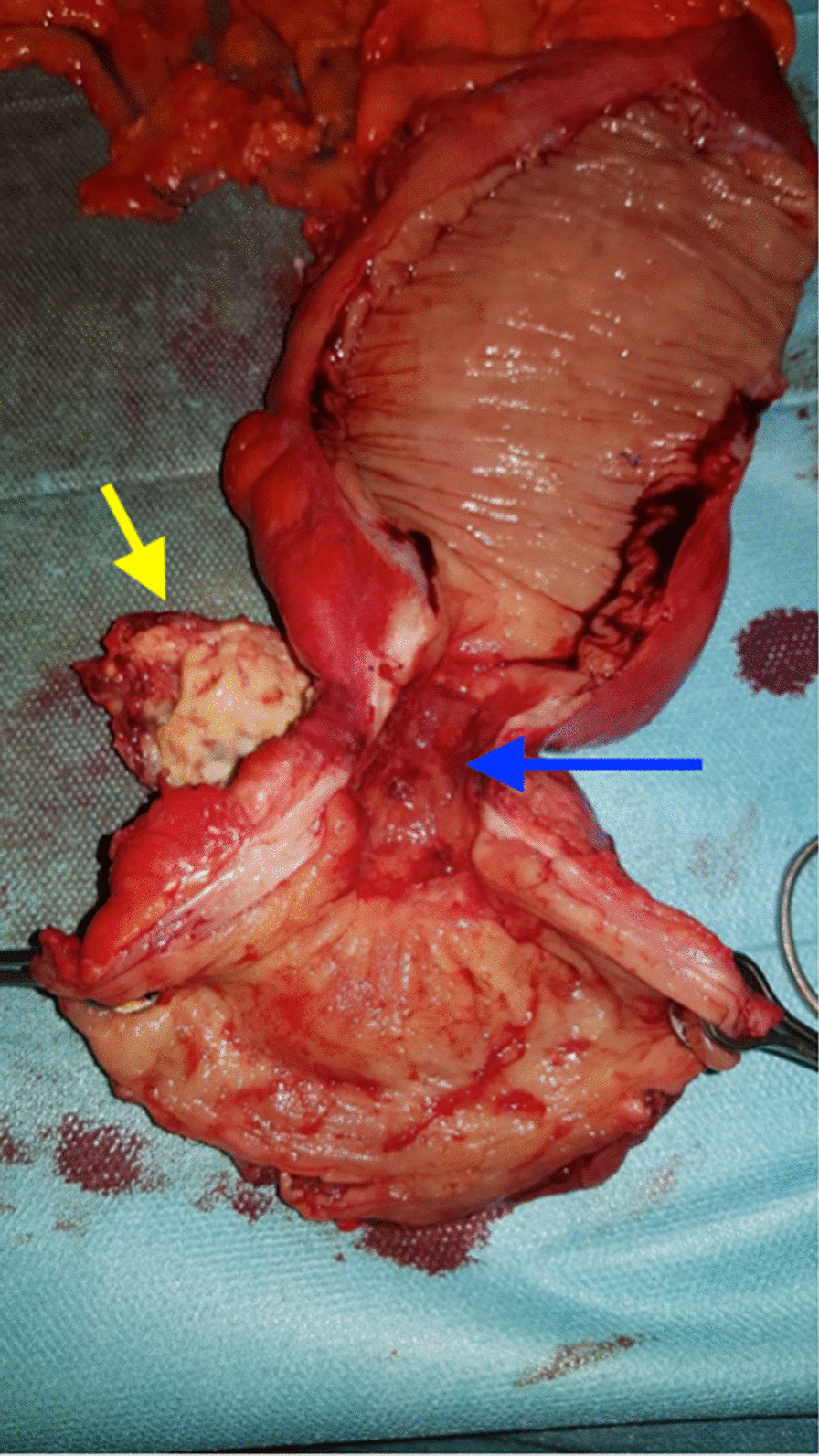
Resection specimen showing tumor stenosis (blue arrow) and actinomycotic pseudotumor (red arrow)

Due to the low location of the cancer and its “local extension,” chemoradiotherapy (CR) administered over a period of 5 weeks was established. An anterior resection of the rectum with low mechanical colorectal anastomosis protected by an ileostomy was performed 10 weeks after the end of the CR. The operation was carried out by laparotomy to minimize the risk of intrusion of the mesorectum. Postoperative follow-up was simple. Pathological study of the specimen noted the complete sterilization of the tumor after CR with the presence of large foci of suppurative necrosis containing actinomycotic grains in the mesorectum; lymph node dissection revealed ten negative nodes (Figure 3). No adjuvant treatment was recommended. However, antibiotic therapy by protected amoxicillin has been established.

## Discussion

Actinomycosis is a slow and misleading granulomatous infection caused by *Actinomyces*, which is Gram-positive, strict anaerobic bacteria. *Actinomyces israelii* is the principal pathogenic species isolated in human infections [1].

These bacteria are saprophytes of humans’ natural cavities, particularly the oral cavity, the digestive tract, and the genital mucosa [2–4]. These bacteria become pathogenic after breaking of the gastrointestinal mucosa following abdominopelvic surgery, infection, or appendicular and diverticular perforation, or during immunosuppression due to diabetes, steroids, or cancer [5, 6]. Once the mucosa is affected, the infection spreads by contiguity, explaining the damage to organs nearby. Hematogenous or lymphatic spread is rare [1, 7]. The abdominal location of actinomycosis represents 20–24% and ranks second after the cervicofacial location (60%); the thoracic location does not exceed 15%. In the abdominal form, the ileocecal region is affected in more than half of cases (65%) [1, 2]. Pelvic actinomycosis can arise from an anal crypt causing anal or rectal fistulas, and can also be caused by intrauterine device or intravaginal foreign body [2, 8].

Actinomycosis evolves insidiously over months or years. It typically gives abscesses that evolve in the long term to the formation of pseudotumors that invade organs close by. The clinical presentation varies depending on the stage of the evolution of the disease; in the acute phase, it can take on the appearance of peritonitis or pelviperitonitis with an infectious syndrome. Later, in the pseudotumor phase, the tumor syndrome is in the foreground with a palpable, ill-defined, and sensitive mass, more or less indurated with a tendency to invade the abdominal wall and to fistulate on the skin [1, 9].

Biology is not very contributory, and hyperleukocytosis with elevation of inflammatory markers can be noted in suppurative forms [10, 11].

Imaging examinations are not specific. Ultrasonography and computed tomography (CT) scan can show a solid or cystic mass, with thick walls, multilocular or not, frequently evoking a neoplastic process [1, 12]. In addition to specifying locoregional extension of the disease, these examinations can guide a percutaneous biopsy for histologic study that confirms the diagnosis when it reveals actinomycotic grains [2, 12, 13].

Bacteriological diagnosis is difficult and must meet a couple of requirements: sampling and seeding done in rigorous aseptic conditions, and anaerobic culture (7–20 days) in special environments [1].

Differential diagnosis arises mainly with cancer, tuberculosis, Crohn’s disease, and amoebiasis or appendicitis [5].

The treatment of actinomycosis is based on antibiotic therapy by high doses of penicillin G administered intravenously (10–20 MIU) for 4–6 weeks followed by oral relay with amoxicillin + clavulanic acid associated to metronidazole for 6–12 months. In the case of an allergy to penicillin, other antibiotics can be used: tetracyclines, macrolides, vancomycin, chloramphenicol, or rifampicin [1, 14].

The particularity of our observation is that the diagnosis of actinomycosis was a fortuitous discovery. Indeed, the extension of the rectal adenocarcinoma in the mesorectum, objectified on imaging tests, and which did not regress after the neoadjuvant CR, was only a mesorectal actinomycotic pseudotumor. This has often been mistaken as a locally advanced cancer with a bad prognosis and risk of incomplete resection. It also was why we opted straight away for a median laparotomy instead of a laparoscopy to minimize the risk of mesorectal break-in.

## Conclusion

The abdominopelvic actinomycosis is a rare pathology, with misleading clinical manifestations, which can often be the cause of differential diagnosis with several diseases including cancer. In our case, the occurrence of mesorectal actinomycosis is probably due to the development of the adenocarcinoma in the rectum and mistaken for a local extension of this cancer.

## Data Availability

Not applicable.
